# Nick sealing of polβ mismatch insertion products by LIG1 and LIG3α during 8-oxoG bypass leads to mutagenic or error-free base excision repair

**DOI:** 10.1016/j.jbc.2025.108540

**Published:** 2025-04-24

**Authors:** Kar Men Lee, Erick Castro, Jacob Ratcliffe, Camden Lerner, Melike Çağlayan

**Affiliations:** Department of Biochemistry and Molecular Biology, University of Florida, Gainesville, Florida, USA

**Keywords:** base excision repair, DNA polymerase β, DNA ligase 1, DNA ligase 3, oxidative DNA damage, 8oxoG, AP-Endonuclease 1

## Abstract

Base excision repair (BER) requires a coordination at the downstream steps involving gap filling by DNA polymerase (pol) **β** and subsequent nick sealing by DNA ligase (LIG) 1 or 3**α**. We previously reported that a failure in DNA ligase function, stemming from an impairment in nick sealing of pol**β** nucleotide insertion products, leads to faulty repair events. Yet, how the fidelity of 8-oxoG bypass by pol**β** affects the efficiency of ligation remains unclear. Here, we show that LIG1 and LIG3**α** seal the resulting nick repair product of pol**β** mutagenic insertion of dATP opposite 8-oxoG, while LIG3**α** exhibits an inability to ligate pol**β** dCTP:8-oxoG insertion product, demonstrating that the identity of BER ligase plays a critical role in repair outcomes at the final step. Furthermore, our results show that a lack of ribonucleotide insertion by pol**β** during 8-oxoG bypass diminishes the repair coordination with both ligases, highlighting the critical role of nucleotide selectivity in maintaining BER accuracy. Finally, our results reveal that AP-Endonuclease 1 (APE1) proofreads nick repair intermediates containing 3′-mismatches or ribonucleotides templating 8-oxoG. Overall, our findings provide a mechanistic insight into how the dual coding potential of the oxidative lesion in -*anti versus* -*syn* conformation could govern error-prone *versus* error-free repair outcomes, leading to deviations in the BER pathway coordination and the formation of deleterious DNA intermediates.

Oxidative stress caused by reactive oxygen species (ROS) such as superoxide and hydroxyl radicals which arise during metabolic processes in cells and by the effect of environmental exposures such as ionizing radiation is the major threat to genome stability (1). The oxidation of DNA bases by ROS can occur directly in the genomic DNA or indirectly within the nucleotide pool (2). Such oxidative lesions can cause mutations or cell death if they are not efficiently repaired (3). Guanine is more susceptible to be oxidized by ROS compared to the other four DNA bases due to having the lowest oxidation potential, and guanine oxidation, a phenomenon that underlies age-related disorders including cancer and neurological diseases, leads to 7,8-dihydro-8-oxo-guanine (8-oxoG) formation (4, 5).

The coding potential of 8-oxoG is dictated by *anti*- and *syn*-conformations of the oxidized base, which plays a pivotal role in determining its mutagenic potential (6). The equilibrium between these conformations can be influenced by various factors such as cellular environment and presence of specific DNA polymerase (7, 8, 9, 10). In the -*syn* conformation, 8-oxoG pairs with adenine through Hoogsteen base pair leading to a G-T transversion mutation during replication, especially in the cancer genome (11). This is caused by spatial arrangement of the -*syn* conformation of 8-oxoG that closely resembles that of thymine, prompting the polymerase to insert adenine, which constitutes a significant source of oxidative damage-related mutagenesis in cells (9, 12, 13). Conversely, 8-oxoG in the -*anti* conformation can pair with cytosine through traditional Watson-Crick base pairing as an unoxidized guanine, forming a non-mutagenic DNA lesion (9, 14, 15).

The major defense mechanism against the accumulation of 8-oxoG is base excision repair (BER), preventing the mutagenic and lethal consequences of the oxidative DNA damage generated by endogenous ROS and environmental toxicants (16, 17, 18). The BER pathway involves a series of sequential enzymatic steps and requires a tightly coordinated function of the repair proteins (19, 20). The repair pathway begins with a lesion-specific DNA glycosylase that removes a damaged base, resulting in an abasic or AP-site in double-stranded DNA (21). This lesion is then recognized by AP endonuclease 1 (APE1), which cleaves the phosphodiester backbone, generating a repair intermediate with a single-strand break containing a 3′-OH and five′-deoxyribose phosphate (5′-dRP) group (22). DNA polymerase (pol) β then removes the 5′-dRP group and catalyzes DNA synthesis by incorporating a single nucleotide into a gap repair intermediate, resulting in the formation of a nick with 3′-OH and 5′-PO_4_ ends (23). Finally, DNA ligase (LIG), 1 or LIG3α seals the resulting nick repair product by catalyzing a phosphodiester bond formation to join DNA ends to complete the BER pathway (24). The repair of 8-oxoG mainly by DNA glycosylases in prokaryotic and eukaryotic cells has been extensively studied (25). 8-oxoguanine DNA glycosylase-1 (OGG1) is the primary enzyme that specifically recognizes and excises 8-oxoG when paired with cytosine by flipping the damaged base out of the DNA helix and cleaving the N-glycosidic bond, creating an AP site that is then further processed by subsequent proteins in the repair pathway (26). When 8-oxoG escapes repair, there is a high probability that replication will result in adenine insertion. Thus, the cell also codes for a DNA glycosylase, MutY homolog (MYH or MUTYH), which recognizes and removes adenine paired with 8-oxoG that adopts the mutagenic -*syn* conformation by excising the mispaired adenine (27). Additionally, MutT in bacteria and MutT homolog 1 (MTH1) in humans hydrolyze oxidized nucleotides, particularly 8-oxoGTP, to its monophosphate form, thereby preventing their incorporation into the genome by DNA polymerases (28, 29). Together, the coordinated actions of OGG1, MYH, and MTH1 effectively prevent accumulation of oxidative damage from causing mutations and contribute to maintaining the fidelity of the genetic code (30, 31).

During the gap filling step of the BER pathway, polβ can incorporate 8-oxodGTP in both -*syn* and -*anti* conformations opposite adenine and cytosine, respectively (32). We previously reported that the nick repair intermediate with 3′-8oxodGMP inserted by polβ confounds the next ligation step by LIG1 and LIG3α, resulting in ligase failure and formation of abortive ligation products with 5′-adenylate (33, 34). When polβ encounters the oxidative damage lesion on the template position, the polymerase incorporates dATP or dCTP opposite 8-oxoG in one nucleotide gap DNA as shown in kinetics studies of the enzyme (35, 36, 37, 38, 39). Particularly, the processing of polβ dATP:8-oxoG insertion products could lead to mutagenic repair that can be a cellular burden during times of elevated metabolic or environmental stress (36, 40, 41). Furthermore, time-resolved X-ray crystallography of polβ demonstrated structural snapshots that were captured at specific time points of phosphodiester bond formation in the process of nucleotide incorporation during 8-oxoG bypass. This allowed to visualize both Watson-Crick (*anti*-8-oxoG:*anti*-dCTP) and Hoogsteen (*syn*-8-oxoG:*anti*-dATP) base pairing, which was facilitated by a third Mg^2+^ ion as a stabilizing reaction intermediate (42). These studies demonstrate how the fidelity of polβ 8-oxoG bypass could govern the repair outcomes, yet it remains unknown how this error-free or error-prone gap filling by polβ could affect the next ligation step.

Due to the higher abundance of ribonucleotide triphosphates (rNTPs) relative to deoxyribonucleotide triphosphates (dNTPs) in human cells, repair and replication polymerases incorporate rNTPs frequently into the genome (43). Polβ exhibits sugar discrimination by utilizing a backbone carbonyl to clash with the 2′-OH of rNTPs through the steric gate residue Y271 (44). It has also been shown that polβ bypasses 8-oxoG more faithfully in the presence of rNTPs than dNTPs (45). Most recently, we reported how the ribonucleotide insertion can adversely impact gap filing function of polβ leading to repair pathway discoordination with the BER ligases (46, 47, 48).

Our previous studies demonstrated the molecular determinants of faithful BER at the downstream steps and have documented cases of BER pathway coordination involving consecutive activities of polβ and DNA ligases (33, 49, 50, 51, 52, 53, 54). We reported a range of deviations, especially in the case of mismatch insertions by polβ opposite undamaged bases in gap DNA, resulting in faulty repair events and formation of deleterious DNA intermediates. Although these studies emphasize the importance of polβ in modulating the repair, it remains unknown how polβ mismatch insertion during 8-oxoG bypass affects the accuracy of the repair pathway coordination at the downstream steps where BER ligases seal the resulting nick product. Particularly, the polβ structures with an incorporated nucleotide (dAMP or dCMP) and template 8-oxoG demonstrated a transition from a closed ternary to open binary conformation facilitated by unique stacking interactions and a third metal ion (42), suggesting that this open binary conformation could enhance a transfer of the resulting nick repair product from polβ dAMP or dCMP insertion during 8-oxoG bypass to DNA ligase for the completion of BER.

In the present study, we questioned the impact of polβ mismatch or ribonucleotide insertions opposite 8-oxoG on the efficiency of DNA ligation by LIG1 and LIG3α. We also interrogated the role of APE1 in the removal of 3′-mismatched base through 3′-5′ exonuclease activity and its coordination with the nick sealing step by LIG1 and LIG3α. We showed the mutagenic ligation by both BER ligases after polβ dATP:8-oxoG insertion, while nick sealing efficiency of LIG3α after polβ inserts dCTP:8-oxoG was drastically reduced, demonstrating that ambiguous coding potential and the identity of BER ligase modulate error-prone *versus* error-free repair outcomes at the final steps. Furthermore, the repair pathway coordination between polβ and BER ligases was diminished in the presence of ribonucleotide mismatches templating 8-oxoG. Finally, our results showed that APE1 removes 3′-ribonucleotides more efficiently than 3′-mismatches when paired with 8-oxoG.

Overall, our findings provide a mechanistic insight into how 8-oxoG bypass by polβ impacts next ligation step by LIG1 *versus* LIG3α, depending on the dual coding potential of the oxidative lesion in -*anti versus* -*syn* conformation. We demonstrate the deviations from canonical repair pathway coordination after polβ dNTP *versus* rNTP mismatch incorporations with BER ligases. Our results also highlight the importance of functional interplay between APE1, polβ, LIG1, and LIG3α at the downstream steps in ensuring effective repair.

## Results

### Ligation of pol**β** mismatch insertion products during 8-oxoG bypass by LIG1 and LIG3**α**

**Figure 1:**
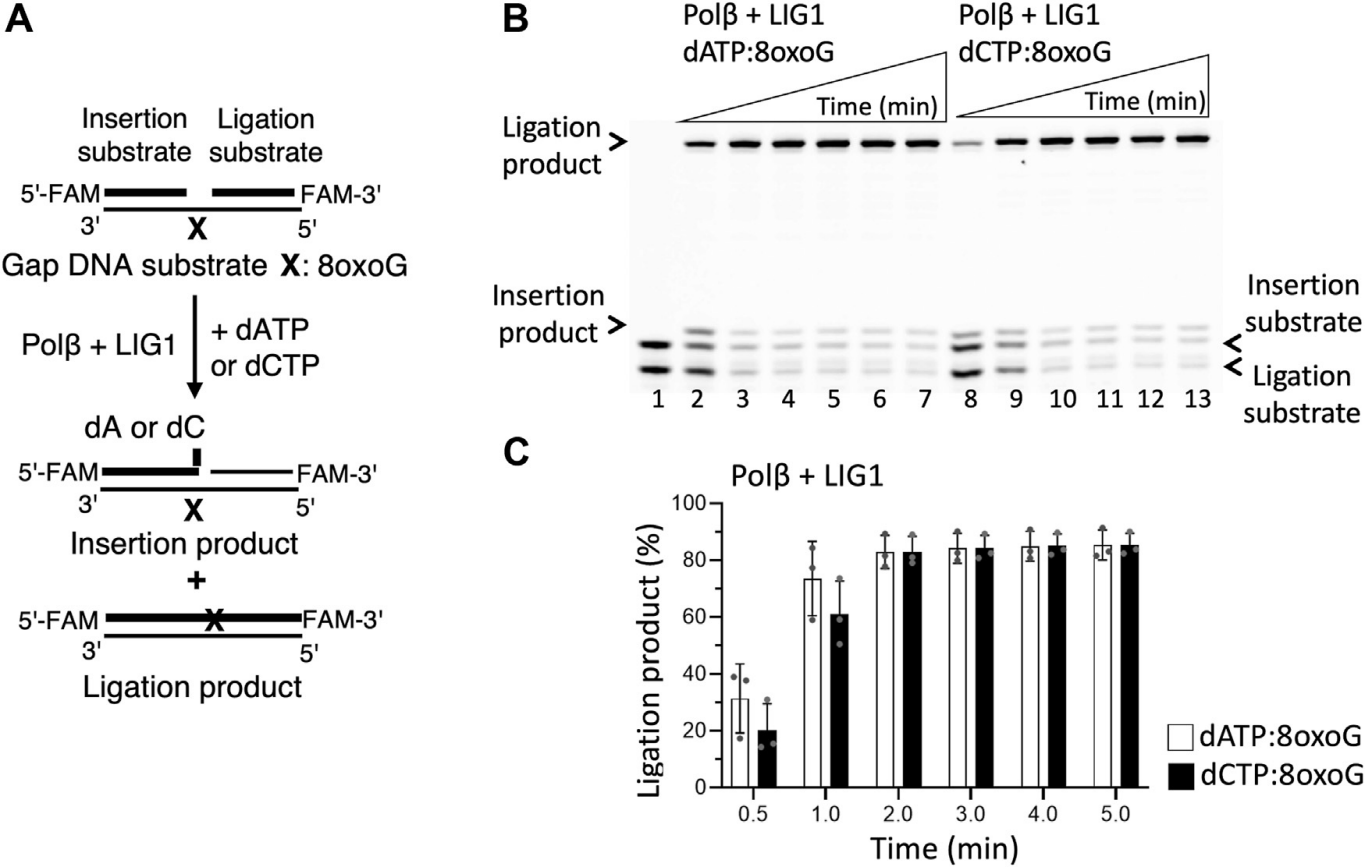
**Ligation of polβ8-oxoG bypass products by LIG1**. *A*, scheme shows reaction substrate and products observed in the coupled assays including polβ and LIG1 to test dNTP (dATP or dCTP) insertion opposite template 8-oxoG in gap DNA. *B*, line 1 is the negative enzyme control of the one nucleotide gap DNA substrate with template 8-oxoG. Lanes 2 to 7 and 8 to 13 are the ligation products of polβ dATP and dCTP insertions opposite 8-oxoG, respectively, by LIG1 and correspond to time points of 0.5, 1, 2, 3, 4, and 5 min. *C*, graph shows time-dependent changes in the amount of ligation products and the data represent the average of three independent experiments ± SD.

**Figure 2:**
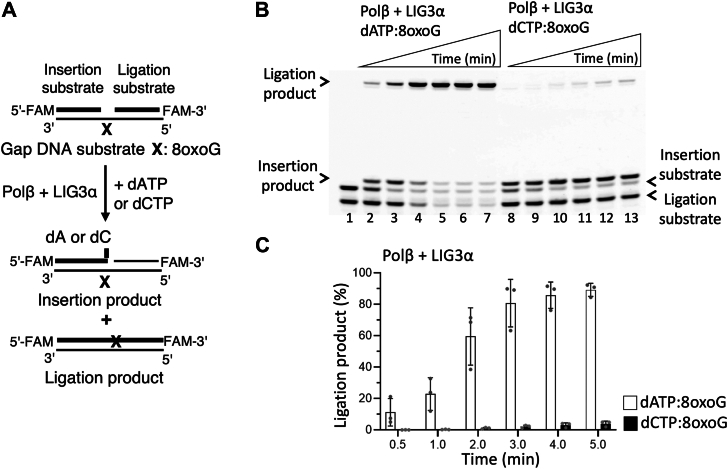
**Ligation of polβ8-oxoG bypass products by LIG3α**. *A*, scheme showing reaction substrate and products observed in the coupled assays, including polβ and LIG3α to test dNTP (dATP or dCTP) insertion opposite template 8-oxoG in gap DNA. *B*, line 1 is the negative enzyme control of the one nucleotide gap DNA substrate with template 8-oxoG. Lanes 2 to 7 and 8 to 13 are the ligation products of polβ dATP and dCTP insertions opposite 8-oxoG, respectively, by LIG3α and correspond to time points of 0.5, 1, 2, 3, 4, and 5 min. *C*, graph shows time-dependent changes in the amount of ligation products and the data represent the average of three independent experiments ± SD.

We first investigated dATP and dCTP mismatch insertion by polβ during 8-oxoG bypass and subsequent nick sealing by LIG1 and LIG3α (Figs. 1*A* and 2*A*). Polβ can insert dATP:8-oxoG and dCTP:8-oxoG into a gap DNA (Figs. S1*A* and S2*A*). In the presence of polβ and LIG1, the ligation products after both insertions were obtained (Fig. 1*B*). However, LIG3α was only able to seal the resulting nick product of polβ dATP:8-oxoG insertion, which was relatively less efficient when compared to that of LIG1 (Fig. 1*B versus* 2*B*, lanes two–7). Interestingly, no ligation product was observed after polβ dCTP:8-oxoG insertion by LIG3α, when compared to an efficient ligation by LIG1 (Fig. 1*B versus* 2*B*, lanes 8–13).

The comparisons of ligation products by the BER ligases demonstrated that the nick sealing by LIG1 was similar after both polβ dATP:8-oxoG and dCTP:8-oxoG insertions, and this ligation efficiency is significantly reduced by LIG3α in the case of polβ dCTP:8-oxoG insertion (Figs. 1*C* and 2*C*). Similarly, the nick sealing profile after polβ dATP:8-oxoG insertion by LIG1 *versus* LIG3α showed no significant difference, while there was ∼80-fold more efficient ligation by LIG1 over LIG3α after polβ dCTP:8-oxoG insertion (Fig. S3). In the control experiments, we confirmed the ligation of polβ correct dGTP:C insertion products by both BER ligases (Fig. S4*A*).

### Impact of pol**β** active site mutations on the ligation efficiency of 8-oxoG bypass products

X-ray structures of polβ binary complexes with an incoming dATP and dCTP opposite template 8-oxoG of a gap DNA reported the importance of polβ active site residues Lys(K)280 and Arg(R)283 for stabilization of the mutagenic -*syn* and non-mutagenic -*anti* conformations of 8-oxoG base pairing at the template-primer terminus (36, 40, 41). To further characterize the repair pathway coordination during polβ 8-oxoG bypass in both conformations, and to investigate the impact of mutations at these critical active sites that modulate the fidelity of the polymerase, we generated polβ K280A and R283K mutants and compared the ligation efficiency after mismatch insertions as described above for wild-type enzyme.

We observed dATP:8-oxoG and dCTP:8-oxoG insertions by polβ K280A, the efficiency of dCTP:8-oxoG insertion was significantly reduced by the effect of R283K mutation, that was not able to insert dATP:8-oxoG (Figs. S1, *B* and *C* and S2, *B* and *C*). In the presence of polβ K280A mutant and LIG1, we observed the ligation of dATP:8-oxoG and dCTP:8-oxoG insertion products at similar efficiency (Fig. S5*A*). In the presence of polβ K280A mutant and LIG3α, this efficiency for converting dATP:8-oxoG insertion to ligation products was slightly lower than that of LIG1 and no ligation product was observed following dCTP:8-oxoG insertion (Fig. S5*B*). Overall, these results demonstrate wild-type level of ligation efficiency by both BER ligases after mismatch insertions by polβ K280A mutant (Fig. S5, *C* and *D*).

However, we showed that the polβ R283K mutation adversely impacts the ligation after mismatch insertions (Fig. S6, *A* and *B*). We only observed nick sealing by LIG1 after dCTP:8-oxoG insertion and there was a time-dependent increase in ligation product after dCTP:8-oxoG insertion by LIG1 (Fig. S6*C*). Conversely, the ligation efficiency of LIG3α was significantly reduced when compared to LIG1 for both insertions by polβ R283K mutant, showing a ∼60-fold difference (Fig. S6*D*). These results demonstrate that the mutations at the polβ active site affects nick sealing efficiency depending on the type of the BER ligase and mismatch being inserted during 8-oxoG bypass (Figs. S7–S8). In the control experiments, we observed differences in the ligation efficiency after a correct dGTP:C insertion by polβ active site mutants K280A and R283K (Fig. S4, *B* and *C*).

In addition to the polβ active site mutants, we also questioned how cancer-associated mutations could affect the efficiency of downstream BER steps during 8-oxoG bypass. Polβ E288K, a colon tumor variant, exhibits an enhanced mutagenesis due to a loss of fidelity although it shows similar gap DNA binding affinity with wild-type enzyme (55). Polβ E288K can insert dATP:8-oxoG and dCTP:8-oxoG at similar efficiency (Figs. S1*D* and S2*D*). In the presence of LIG1, we observed an efficient ligation after both insertions, which was relatively less in the presence of LIG3α after dATP:8-oxoG insertion (Fig. S9, *A* and *B*), and no ligation product was observed after dCTP:8-oxoG insertion as compared in the amount of ligation products by both BER ligases (Fig. S9, *C* and *D*). The results with polβ E288K variant was found to be similar with wild-type enzyme (Figs. S10 and S11), suggesting that 8-oxoG-mediated mutagenesis during BER could be a contributor of the sequence specific mutator phenotype of polβ E288K variant as shown both *in vitro* and *in vivo* studies (56). We observed nick sealing of correct nucleotide insertion products by polβ E288K mutant in the control experiments (Fig. S4*D*).

Overall comparisons showed that the mutations at the polβ active site affect gap filling ability of the wild-type enzyme depending on both the type of mismatch being inserted and the dual potential code of 8-oxoG in -*anti versus -syn* conformation. When compared with wild-type enzyme, we observed similar insertion efficiencies for dATP and dCTP mismatches by E288K mutant, while there was a drastic decrease by the effect of R283K mutation (Figs. S12 and S13). Yet, there was no significant difference in the gap DNA binding affinities of the polβ active site mutants, which were found similar with that of wild-type (Fig. S14). This suggests that subsequent nick sealing by LIG1 and LIG3α during 8-oxoG bypass by polβ relies on the perturbations at the polymerase active site and resulting changes in its mismatch insertion efficiency.

### Impact of pol**β** ribonucleotide incorporation during 8-oxoG bypass on the efficiency of nick sealing by BER ligases

**Figure 3:**
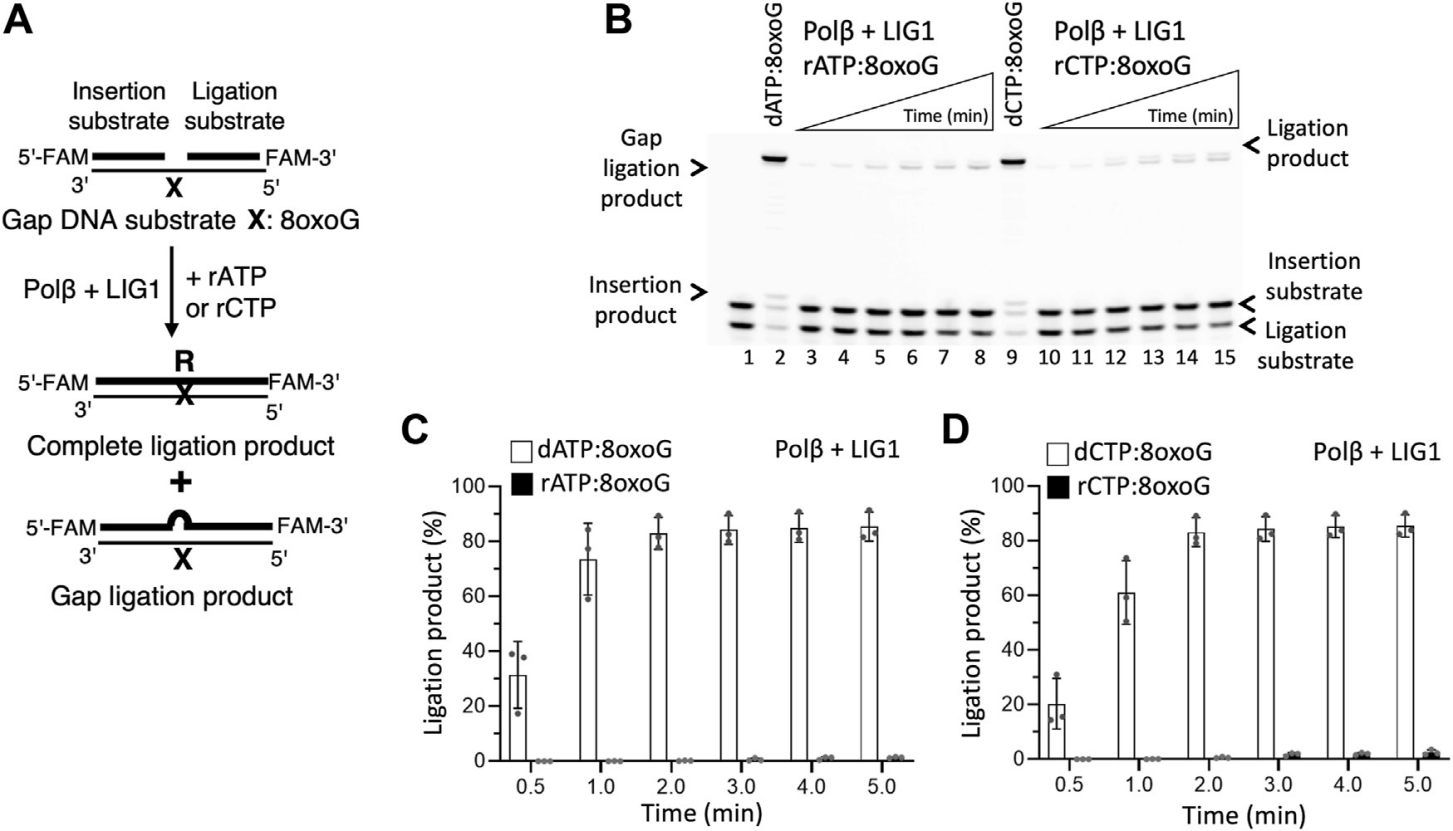
**Ligation of polβ ribonucleotide insertion products during8-oxoG bypass by LIG1**. *A*, scheme shows reaction substrate and products observed in the coupled assays including polβ and LIG1 to test rNTP (rATP or rCTP) insertion opposite template 8-oxoG in gap DNA. *B*, line 1 is the negative enzyme control of the one nucleotide gap DNA substrate with template 8-oxoG. Lanes 2 and 9 are ligation products of polβ dATP:8-oxoG and dCTP:8-oxoG insertions, respectively, by LIG1. Lanes 3 to 8 and 10 to 15 are the ligation products of polβ rATP:8-oxoG and rCTP:8-oxoG insertions, respectively, by LIG1, and correspond to time points of 0.5, 1, 2, 3, 4, and 5 min. *C* and *D*, graphs show time-dependent changes in the amount of ligation products and the data represent the average of three independent experiments ± SD.

**Figure 4:**
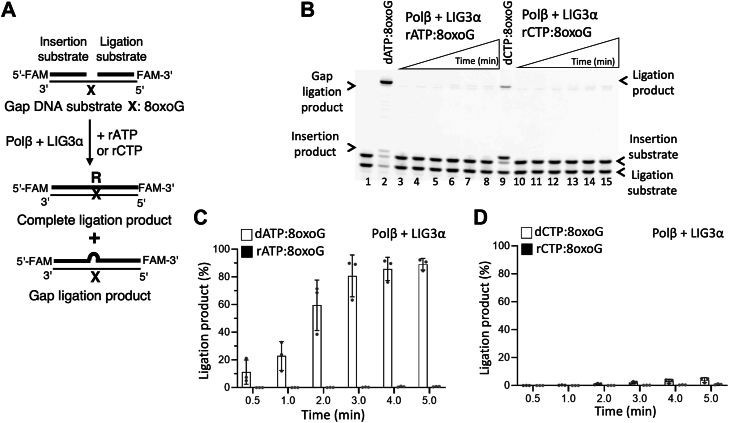
**Ligation of polβ ribonucleotide insertion products during8-oxoG bypass by LIG3α**. *A*, scheme shows reaction substrate and products observed in the coupled assays including polβ and LIG3α to test rNTP (rATP or rCTP) insertion opposite template 8-oxoG in gap DNA. *B*, line 1 is the negative enzyme control of the one nucleotide gap DNA substrate with template 8-oxoG. Lanes 2 and 9 are ligation products of polβ dATP:8-oxoG and dCTP:8-oxoG insertions, respectively, by LIG3α. Lanes 3 to 8 and 10 to 15 are the ligation products of polβ rATP:8-oxoG and rCTP:8-oxoG insertions, respectively, by LIG3α, and correspond to time points of 0.5, 1, 2, 3, 4, and 5 min. *C* and *D*, graphs show time-dependent changes in the amount of ligation products and the data represent the average of three independent experiments ± SD.

To comprehensively elucidate the impact of mismatch insertions by polβ bypassing 8-oxoG on the repair pathway coordination with the BER ligases, we then questioned how rNTP incorporation opposite 8-oxoG could affect the next nick sealing step (Figs. 3*A* and 4*A*). Polβ fails to insert rATP:8-oxoG and rCTP:8-oxoG (Figs. S1*E* and S2*E*). In the presence of LIG1 or LIG3α, our results demonstrated no ligation at all (Figs. 3*B* and 4*B*). We only obtained a gap ligation product by LIG1, which refers to the ligation of one nucleotide gap substrate itself as shown by the difference in size with a complete ligation product after polβ dATP:8-oxoG (Fig. 3*B*, line 2 *versus* lanes 3–8) or dCTP:8-oxoG (Fig. 3*B*, line 9 *versus* lanes 10–15) insertions. These results demonstrated that the nick sealing efficiency after polβ dNTP *versus* rNTP mismatch insertions is drastically different (Figs. 3, *C* and *D* and 4, *C* and *D*), suggesting that 8-oxoG bypass by polβ is dictated by the nature of an incoming nucleotide and identity of the sugar, and thereby, promoting insertion of dATP in *syn*-8-oxoG conformation by polβ primarily leads to mutagenic ligation of the resulting nick product at the final steps of the BER pathway.

### Interplay between APE1 and BER ligases during the removal or ligation of 3′-mismatches from the nick repair intermediates containing template 8-oxoG

**Figure 5:**
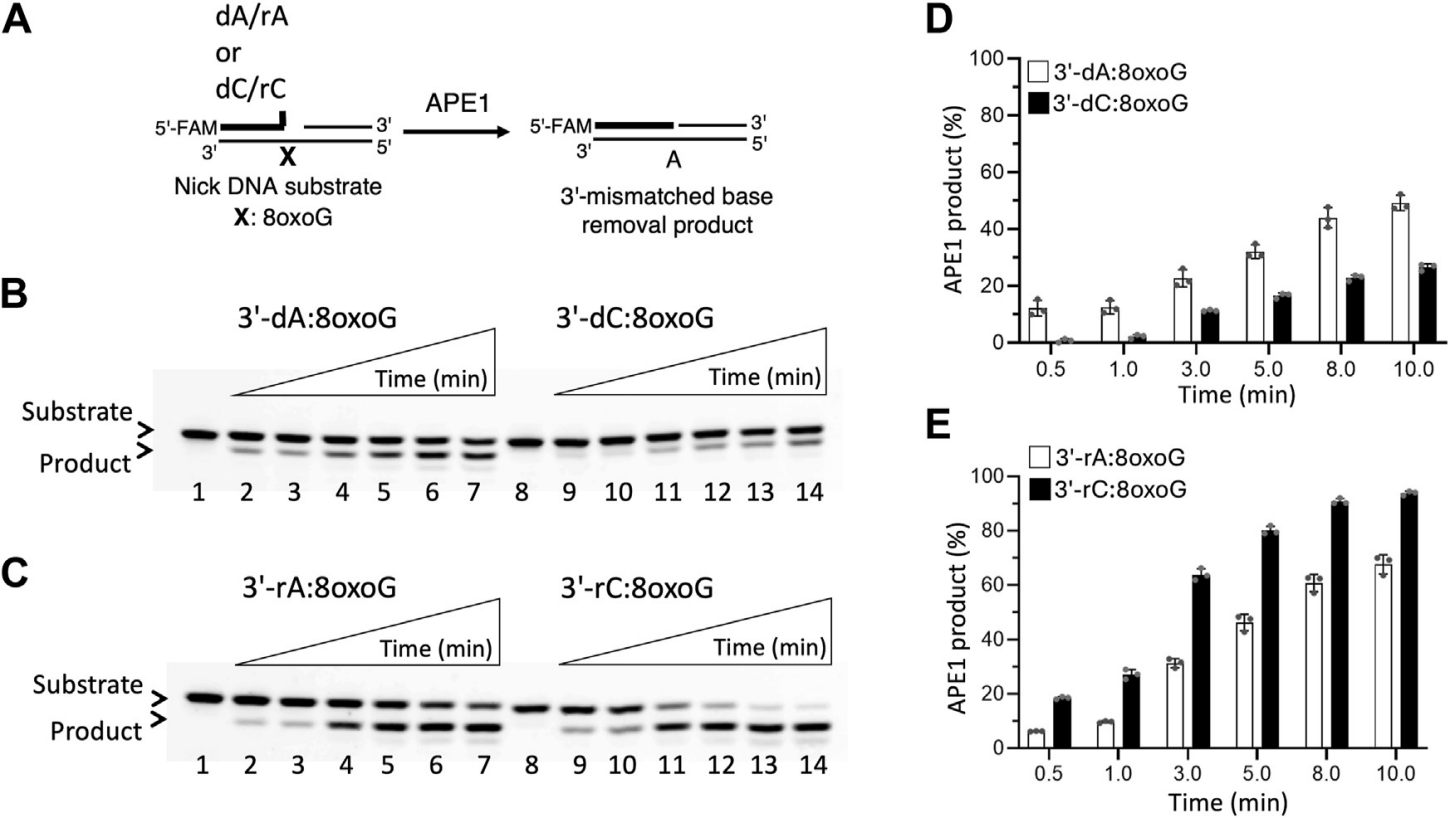
**Mismatch removal by APE1 from the nick substrates with template8-oxoG**. *A*, scheme shows reaction substrate and products observed in the exonuclease assays including APE1 to test the removal of 3′-dA/dC or 3′-rA/rC from the nick DNA substrate containing template 8-oxoG. *B*, lanes 1 and 8 are the negative enzyme controls of the nick DNA substrates with 3′-dA:8-oxoG and 3′-dC:8-oxoG, respectively. Lanes 2 to 7 and 9 to 14 are the removal of 3′-dA and 3′-dC mismatches from nick DNA substrates with 3′-dA:8-oxoG and 3′-dC:8-oxoG, respectively, and correspond to time points of 0.5, 1, 3, 5, 8, and 10 min. *C*, lanes 1 and 8 are the negative enzyme controls of the nick DNA substrates with 3′-rA:8-oxoG and 3′-rC:8-oxoG, respectively. Lanes 2 to 7 and 9 to 14 are the removal of 3′-rA and 3′-rC mismatches from nick DNA substrates with 3′-rA:8-oxoG and 3′-rC:8-oxoG, respectively, and correspond to time points of 0.5, 1, 3, 5, 8, and 10 min. *D* and *E*, graphs show time-dependent changes in the amount of mismatch (*D*) and ribonucleotide (*E*) removal products by APE1. The data represent the average of three independent experiments ± SD.

To comprehensively elucidate the molecular determinants of BER accuracy during polβ 8-oxoG bypass at the downstream steps, we finally investigated the proofreading activity of APE1 and its coordination with the BER ligases (Fig. 5*A*).

Our results demonstrated that APE1 can remove dA or rA and dC or rC mismatches from the 3′-end of nick DNA substrates containing template 8-oxoG (Fig. 5, *B* and *C*). The removal of 3′-mismatches was less efficient than that of 3′-ribonucleotides (Fig. 5, *D* and *E*). In the presence of APE1 and the BER ligases, the products of mismatch removal by APE1 and gap ligation after the mismatch removal are accumulated along with complete ligation products (Fig. S15). In the presence of nick DNA substrates containing 3′-ribonucleotide and template 8-oxoG, our results showed abundant gap ligation products in the presence of 3′-rA:8-oxoG and 3′-rC:8-oxoG, suggesting that both BER ligases can attempt to ligate gap repair intermediate after more efficient removal of 3′-ribonucleotides by APE1 (Fig. S16).

## Discussion

Genomic DNA is constantly exposed to various endogenous sources and exogenous agents that generate base modifications (1). Among the most frequently formed DNA lesions created by oxidative stress, 8-oxoG not only arises spontaneously during normal cellular processes but is also generated by exposure to environmental sources such as UV radiation (2, 3). The 8-oxoG lesion in the template strand could result in a high frequency of misincorporation by DNA polymerases when paired with either correct cytosine or incorrect adenine during replication (4, 5, 6). Structural and kinetics studies have reported the reduced nucleotide selectivity of DNA polymerases at varying degrees across families when they encounter 8-oxoG relative to unmodified template guanine (7, 8, 9, 10). This is mainly because of DNA polymerase-specific variations in the ratio of dC:dA insertion opposite 8-oxoG and interactions at the polymerase active site in different conformations (9, 13, 14, 15).

**Figure 6:**
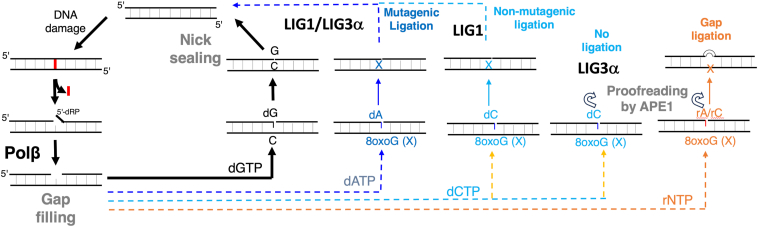
**Working model of the present study**. Illustration of the downstream steps of the BER pathway involving gap filling by polβ and subsequent nick sealing by LIG1 or LIG3α. In the presence of the correct nucleotide insertion by polβ (*i*.*e*., dGTP:C), the nick repair product with 3′-OH and 5′-PO_4_ ends can be efficiently ligated by both BER ligases. In the presence of 8-oxoG in -*syn* conformation, polβ bypassing the oxidative lesion by insertion of dATP mismatch leads to mutagenic ligation of the resulting nick repair product by both LIG1 and LIG3α. Polβ dCTP insertion opposite 8-oxoG results in error-free ligation by LIG1, while the resulting nick repair product cannot be sealed by LIG3α. In the presence of ribonucleotides, the inability of polβ to insert rNTP opposite 8-oxoG results in the ligation of gap repair intermediate and the formation of single deletion mutagenesis products. APE1, *via* its proofreading function, can remove 3′-mismatches and 3′-ribonucleotides from nick repair intermediates containing 8-oxoG on a template position, thereby providing a fidelity checkpoint at the final steps.

As the major cellular defense mechanism against oxidative DNA damage is BER (18), in the present study, we aimed to understand how the fidelity of 8-oxoG bypass by polβ in -*anti versus syn* conformation could affect downstream steps involving final ligation of nick repair intermediate. Considering the insights gained from the current study, we propose a working model (Fig. 6) that illustrates how polβ mismatch or ribonucleotide insertion opposite 8-oxoG could impact the efficiency of subsequent nick sealing by LIG1 and LIG3α. We demonstrated that polβ inserting dATP in error-prone -*syn* conformation results in mutagenic ligation by both BER ligases, leading to futile repair. However, we observed significantly diminished ligation by LIG3α after polβ dCTP:8-oxoG insertion, demonstrating a substrate preference of BER ligase at the final step against polβ nick insertion products containing an 8-oxoG lesion on template position in *anti*- *versus* -*syn* conformation. This could provide a fidelity checkpoint for DNA-end processing enzymes, such as APE1, for proofreading 3′-mismatched bases opposite 8-oxoG from nick repair intermediates.

The ternary complex structures of polβ revealed that K280 side chain is flexible and can adopt multiple conformations (37, 38, 39). Our results demonstrated wild-type level of ligation efficiency by both BER ligases in the case of mutagenic -*syn* conformation of template 8-oxoG in a Hoogsteen base pair with an incoming dATP by polβ K280A active site mutant. Furthermore, X-ray structures of polβ demonstrated that the Hoogsteen base pairing itself stabilizes -*syn* conformation of 8-oxoG, but the enzyme also stabilizes -*syn* conformation using hydrogen bonding between the O8 of 8-oxoG and the R283 side chain, along with an adjustment in the phosphodiester backbone of the template strand (36, 39, 40, 41). Accordingly, we observed a complete ablation of mutagenic nick sealing after dATP:8-oxoG insertion by polβ R283K mutant, which is independent of BER ligase. This could be due to an increased fidelity opposite 8-oxoG by the effect of R283K mutation. Overall, these results provide an insight into the functional interplay between polβ and LIG1/LIG3α in the case of perturbations, particularly due to the amino acid substitutions at the critical active side residues that affect the fidelity of polβ and its lesion bypass specificity leading to adjustments in the position of 3′-OH and 5′-PO_4_ ends of the resulting nick repair intermediate requiring ligation by BER ligases at the final step. Further structural studies are required to better understand the ligase/nick DNA interactions at atomic resolution to elucidate how the ligase active site (LIG1 *versus* LIG3α) engages distinctly with nick complexes containing 8-oxoG on the template position depending on -*anti* or -*syn* conformation.

X-ray repair cross-complementing protein 1 (XRCC1), as a scaffolding factor and another essential protein involved in the maintenance of genome stability, interacts with BER proteins at early and later steps of the pathway to enhance repair efficiency and to prevent labile intermediates form being released (57, 58). Chinese ovary cell lines mutated in XRCC1 have a diminished capacity to initiate the repair of AP sites (59). Through its NTD and BRCT1 domains, XRCC1 makes stable protein complexes with polβ and LIG3α, respectively (60, 61). In our previous studies, we reported the role of XRCC1 in stabilizing repair complexes with both downstream enzymes and facilitating ligation of polβ nucleotide insertion products by LIG3α at the final step (53, 62). However, in mitochondria, LIG3α appears to act independently of XRCC1, as the scaffolding factor is not present in this organelle, and the critical biological role of LIG3α is to maintain mtDNA integrity in response to oxidative stress-induced damage, which is not XRCC1-dependent. (63, 64). We suggest that the ligation efficiency following polβ 8-oxoG bypass after mutagenic dATP mismatch insertion by LIG3α could be XRCC1 independent.

In addition to dNTP mismatches, we also demonstrated how ribonucleotide challenge could affect the ability of polβ bypassing the oxidative lesion and subsequent nick sealing step. Our results showed a discrimination against rNTP incorporation opposite 8-oxoG by polβ as the key factor in preventing the ligation of nick repair intermediates. Overall results highlight a difference in the outcomes of the BER pathway coordination between polβ dNTP *versus* rNTP insertions coupled to ligation by LIG1 and LIG3α, specifically halting ligation when ribonucleotides are inserted across from template 8-oxoG and resulting in the ligation of one nucleotide gap repair intermediate (Fig. 6). Despite the ribonucleotide’s ability to code for bases, a single nucleotide deletion could be more detrimental than an embedded one due to potential disruptions in the DNA sequence. We previously reported the physical interaction and importance of functional interplay between APE1 and BER ligases, LIG1 and LIG3α, for efficient repair (49). Accordingly, our results highlight this fidelity check point for BER accuracy at the downstream steps where APE1 removes 3′-mismatches or 3′-ribonucleotides from nick repair intermediate containing template 8-oxoG (Fig. 6). In summary, our study contributes to understanding how mutagenic *versus* error-free 8-oxoG lesion bypass could modulate the repair outcomes and how the structural conformations that polβ exhibits depending on balance between the -*anti* and the -*syn* conformations of 8-oxoG and the identity of an incoming nucleotide (dNTP *versus* rNTP) to be incorporated opposite the oxidative lesion could impact the repair efficiency at the downstream steps of BER pathway.

Cell studies have been reported that the 8-oxoG repair pathway is coordinated by MUTYH glycosylase and X-family DNA polymerases. For example, by immunofluorescence experiments, a specific recruitment of MUTYH, polλ, polβ, LIG1 and LIG3α from human cell extracts to A:8oxoG DNA, but not to undamaged DNA, was reported in cells exposed to ROS (65). Similarly, the regulation of oxidative DNA damage repair by polβ and MutYH by cross-talk of phosphorylation and ubiquitination *in vitro* and *in vivo* was shown for repair of A:8-oxo-G mispairs (66, 67). We also previously reported that DNA ligase inhibition enhances hypersensitivity to oxidative stress-inducing agent KBrO_3_ in polβ^+/+^ wild-type mouse embryonic fibroblasts cells more than polβ^−/−^ null cells (52). Future studies will be required to determine the repair of A:8-oxo-G mispairs in response to oxidative stress and in coordination with DNA glycosylases processing the oxidative lesion in those model BER cells.

## Experimental procedures

### Protein purifications

DNA polymerase (pol) β (pET-28a) protein was purified as described (46, 47, 48, 49, 50, 51, 52, 53, 54). Briefly, the protein was overexpressed in BL21(DE3) *E*. *coli* cells in Lysogeny Broth (LB) media at 37 °C for 8 h and induced with 0.5 mM isopropyl β-D-thiogalactoside (IPTG). The cells were then grown overnight at 16 °C. After cell lysis at 4 °C by sonication in the lysis buffer containing 1X PBS (pH 7.3), 200 mM NaCl, 1 mM Dithiothreitol (DTT), and complete protease inhibitor cocktail. The lysate was pelleted at 16,000 x rpm for 1 h and then clarified by centrifugation and filtration. The supernatant was loaded on a HisTrap HP column and purified with an increasing imidazole gradient (0–300 mM) elution at 4 °C. The collected fractions including his-tag polβ protein were then subsequently loaded on a HiTrap Heparin column and eluted with a linear gradient of NaCl up to 1 M. Polβ mutants R283K, E288K and K280A were purified as described for wild-type protein above. DNA ligase (LIG) 3α (pET-24b) protein was purified as described (46, 47, 48, 49, 50, 51, 52, 53, 54). Briefly, the protein was overexpressed in BL21(DE3) *E*. *coli* cells in LB media at 37 °C for 8 h and induced with 0.5 mM IPTG. The cells were harvested, lysed at 4 °C, and then clarified as described above. The supernatant was loaded on a HisTrap HP column and purified with an increasing imidazole gradient (0–300 mM) elution at 4 °C. The collected fractions including his-tag LIG3α protein were then further purified by a Heparin with a linear gradient of NaCl up to 1 M and then finally by a Superdex 200 Increase 10/300 column in the buffer containing 50 mM Tris-HCl (pH 7.0), 500 mM NaCl, 5% glycerol, and 1 mM DTT. DNA ligase (LIG) 1 (pET-24b) protein was purified as described (46, 47, 48, 49, 50, 51, 52, 53, 54). Briefly, the protein was overexpressed in BL21(DE3) *E*. *coli* cells and the cells were harvested, lysed at 4 °C, and then clarified as described above. The supernatant was loaded on a HisTrap HP column and purified with an increasing imidazole gradient (0–300 mM) elution at 4 °C. The collected fractions were then subsequently loaded on a HiTrap Heparin column with a linear gradient of NaCl up to 1 M. His-tag LIG1 protein was then further purified by a Superdex 200 Increase 10/300 column in the buffer containing 50 mM Tris-HCl (pH 7.0), 500 mM NaCl, 5% glycerol, and 1 mM DTT. AP-Endonuclease 1 (APE1) protein (pET-24b) was purified as described (46, 47, 48, 49, 50, 51, 52, 53, 54). Briefly, the protein was overexpressed in BL21(DE3) *E*. *coli* cells and the cells were harvested, lysed at 4 °C, and the supernatant was loaded on a HisTrap HP column as described above. His-tag APE1 protein was purified with an increasing imidazole gradient (0–300 mM) elution and then loaded on a HiTrap Heparin column to further purify as a linear gradient of NaCl up to 1 M, and then finally loaded on a Superdex 200 Increase 10/300 column in the buffer containing 50 mM Tris-HCl (pH 7.0), 500 mM NaCl, 5% glycerol, and 1 mM DTT. All proteins used in this study were dialyzed against storage buffer containing 25 mM TrisHCl (pH 7.4), 100 mM KCl, 1 mM TCEP, and 10% glycerol, concentrated, frozen in liquid nitrogen, and stored at −80 °C in aliquots. Final purity of purified proteins was evaluated on a 10% SDS-PAGE (Fig. S17).

### Pol**β** nucleotide insertion assays

Polβ nucleotide insertion assays were performed as described (46, 47, 48, 49, 50, 51, 52, 53, 54). One nucleotide gap DNA substrates with template 8-oxoG or C were used to test polβ dNTP (dATP or dCTP) or rNTP (rATP or rCTP) insertion opposite 8-oxoG and correct dGTP insertion opposite C (Table S1). The reaction mixture contains 50 mM Tris-HCl (pH 7.5), 100 mM KCl, 10 mM MgCl_2_, 1 mM ATP, 1 mM DTT, 100 μg ml^-1^ BSA, 1% glycerol, dNTP (100 μM), and DNA substrate (500 nM) in the final volume of 10 μl. The reaction was initiated by the addition of polβ (10 nM) and incubated at 37 °C for the time points as indicated in the figure legends. The reaction products were then mixed with an equal amount of gel loading buffer containing 95% formamide, 20 mM EDTA, 0.02% bromophenol blue, and 0.02% xylene cyanol and separated by electrophoresis on 18% Urea-PAGE gel. The gels were finally scanned with a Typhoon PhosphorImager (Amersham Typhoon RGB), and the data were analyzed using ImageQuant software. The nucleotide insertion assays were performed similarly for polβ wild-type and mutants K280A, R283K, and E288K.

### Pol**β** nucleotide insertion coupled to DNA ligation assays

Coupled assays were performed as described (46, 47, 48, 49, 50, 51, 52, 53, 54). One nucleotide gap DNA substrates with template 8-oxoG or C were used to test the ligation efficiency after polβ dNTP (dATP or dCTP) or rNTP (rATP or rCTP) insertion opposite 8-oxoG and polβ correct dGTP insertion opposite C by LIG1 and LIG3α (Table S2). The reaction mixture contains 50 mM Tris-HCl (pH 7.5), 100 mM KCl, 10 mM MgCl_2_, 1 mM ATP, 1 mM DTT, 100 μg ml^-1^ BSA, 1% glycerol, dNTP (100 μM), and DNA substrate (500 nM) in the final volume of 10 μl. The reaction was initiated by the addition of polβ and DNA ligase protein complex (100 nM) and incubated at 37 °C for the time points as indicated in the figure legends. The reaction products were then mixed with an equal amount of gel loading buffer, separated by electrophoresis on 18% Urea-PAGE gel, and the data were analyzed using ImageQuant software as described above. The coupled assays were performed similarly for polβ wild-type and mutants K280A, R283K, and E288K.

### APE1 exonuclease assays

APE1 exonuclease assays were performed as described (46, 47, 48, 49, 50, 51, 52, 53, 54). Nick DNA substrates with preinserted 3′-dA:8-oxoG, 3′-dC:8-oxoG, 3′-rA:8-oxoG, and 3′-rC:8-oxoG were used to test APE1 exonuclease activity for the removal of 3′-mismatched base (dA/rA or dC/rC) from template 8-oxoG (Table S3). The reaction mixture contains 50 mM Tris-HCl (pH 7.5), 100 mM KCl, 10 mM MgCl_2_, 1 mM ATP, 1 mM DTT, 100 μg ml^-1^ BSA, 1% glycerol, and DNA substrate (500 nM) in the final volume of 10 μl. The reaction was initiated by the addition of APE1 alone (1 μM) and incubated at 37 °C for the time points as indicated in the figure legends. The reaction products were then mixed with an equal amount of gel loading buffer, separated by electrophoresis on 18% Urea-PAGE gel, and the data were analyzed using ImageQuant software as described above.

### APE1 exonuclease coupled to DNA ligation assays

Coupled assays were performed as described (46, 47, 48, 49, 50, 51, 52, 53, 54). Nick DNA substrates with preinserted 3′-dA:8-oxoG, 3′-dC:8-oxoG, 3′-rA:8-oxoG, and 3′-rC:8-oxoG were used to test ligation of nick by LIG1 or LIG3α coupled to the removal of 3′-mismatched base (dA/rA or dC/rC) from nick containing template 8-oxoG by APE1 (Table S4). The reaction mixture contains 50 mM Tris-HCl (pH 7.5), 100 mM KCl, 10 mM MgCl_2_, 1 mM ATP, 1 mM DTT, 100 μg ml^-1^ BSA, 1% glycerol, and DNA substrate (500 nM) in a final volume of 10 μl. The reaction was initiated by the addition of APE1 and DNA ligase protein complex (100 nM) and incubated at 37 °C for the time points as indicated in the figure legends. The reaction products were then mixed with an equal amount of gel loading buffer, separated by electrophoresis on an 18% Urea-PAGE gel, and the data were analyzed using ImageQuant software as described above.

### BioLayer interferometry assays for DNA binding measurements

We analyzed gap DNA binding kinetics of polβ (wild-type and mutants) by BioLayer Interferometry (BLI) assays in real time using the Octet QKe system (Fortebio) as described (53, 62). BLI experiments were performed at 20°C in 96-well microplates using DNA containing a single nucleotide gap and 3′-biotin label (Table S5). Streptavidin (SA) biosensors (Fortebio) were used to attach the biotin-labeled DNA. The SA biosensors were hydrated in the kinetics buffer containing 20 mM HEPES (pH 7.4), 200 mM NaCl, 0.5% BSA, 0.05% Tween 20 at 20 °C for 20 min, and were then immersed in gap DNA (40 nM) in the kinetics buffer. After recording an initial baseline in the kinetics buffer (60 s), the sensors with DNA were exposed to polβ at the concentration range as indicated in the figure legends. In all measurements, the affinity constants (K_D_), the association (k_on_) and dissociation (k_off_) rates were calculated using the ForteBio Data Analysis software with 1:1 binding model. The association rate = k_on_ [ligand][analyte] and the dissociation rate = k_off_ [ligand-analyte]. At equilibrium, forward and reverse rates are equal. All images were drawn using Graph Pad Prism 9. BLI assays were performed similarly for polβ wild-type and mutants K280A, R283K, and E288K.

## Data availability

Information and requests of materials used in this research should be directed to Melike Çaglayan (caglayanm@ufl.edu).

## Supporting information

This article contains supporting information.

## Conflict of interest

The authors declare that they have no conflicts of interest with the contents of this article.
