# Supplementary material for: Nick sealing of polβ mismatch insertion products by LIG1 and LIG3α during 8-oxoG bypass leads to mutagenic or error-free base excision repair
Source: J Biol Chem. 2025 Apr 24;301(6):108540. doi: 10.1016/j.jbc.2025.108540 (PMC12152887; doi:10.1016/j.jbc.2025.108540)
Supplement: Supporting Information [file mmc1.pdf]

Nick sealing of polβ mismatch insertion products by LIG1 and LIG3α during 8-oxoG bypass leads to mutagenic or error-free base excision repair

Kar Men Lee, Erick Castro, Jacob Ratcliffe, Camden Lerner, Melike Çağlayan*

Department of Biochemistry and Molecular Biology, University of Florida, Gainesville, FL 32610, USA

*To whom correspondence should be addressed. Tel.: +1 352-294-8383; Email: caglayanm@ufl.edu

Supplementary Information

Supplementary Figures 1-17

Supplementary Tables 1-5

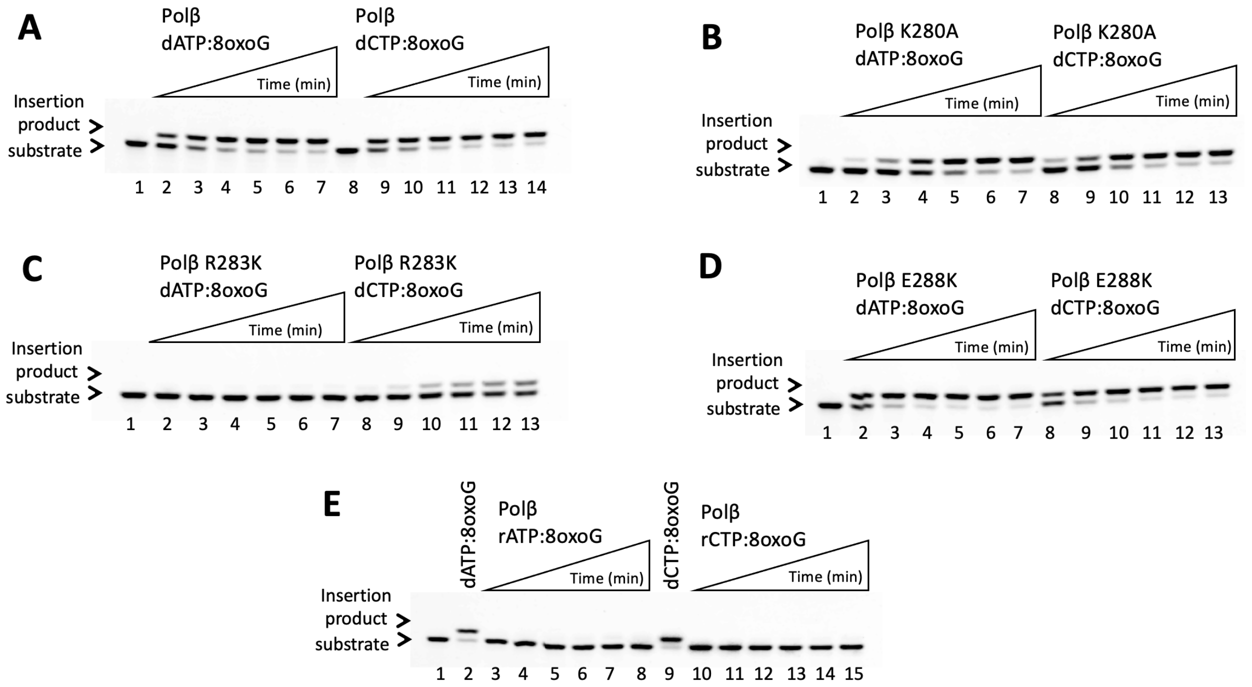

Supplementary Figure 1. Comparison of mismatch nucleotide insertion products by polβ wild-type and mutants during 8-oxoG bypass. (A-D) Lanes 1 are the negative enzyme controls of the one nucleotide gap DNA substrate with template 8-oxoG. Lanes 2-7 and 8-13 are dATP:8-oxoG and dCTP:8-oxoG insertion products by polβ wild-type (A), and mutants K280A (B), R283K (C), E288K (D), and correspond to time points of 0.5, 1, 2, 3, 4, and 5 min. **(E)** Line 1 is the negative enzyme control of the one nucleotide gap DNA substrate with template 8oxoG. Lanes 2 and 9 are dATP:8oxoG and dCTP:8oxoG insertion products, respectively, by polβ wild-type. Lanes 3-8 and 10-15 are rATP:8oxoG and rCTP:8oxoG insertion products by polβ wild-type, respectively.

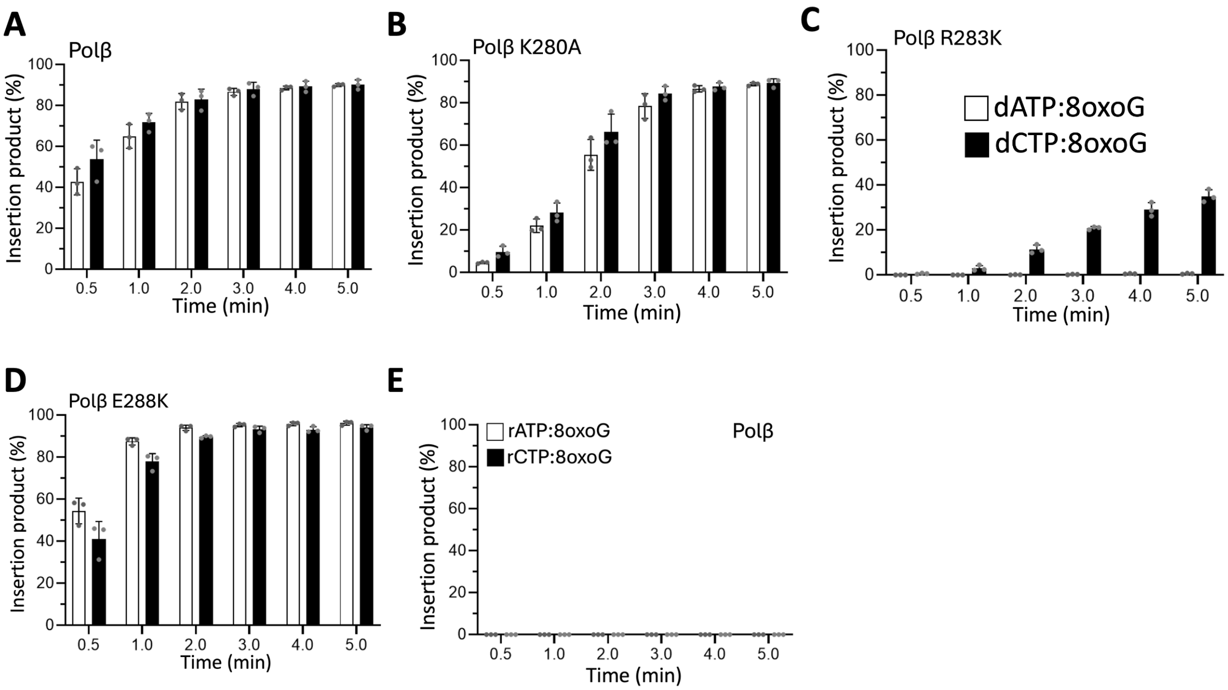

Supplementary Figure 2. Mismatch nucleotide insertion products by polβ wild-type and mutants. (A-D) Graphs show time-dependent changes in the amount of dATP and dCTP insertion products by polβ wild-type (A), K280A (B), R283K (C), and E288K (D) mutants. **(E)** Graph shows time-dependent changes in the amount of rATP and rCTP insertion products by polβ wild-type. The data represent the average of three independent experiments ± SD.

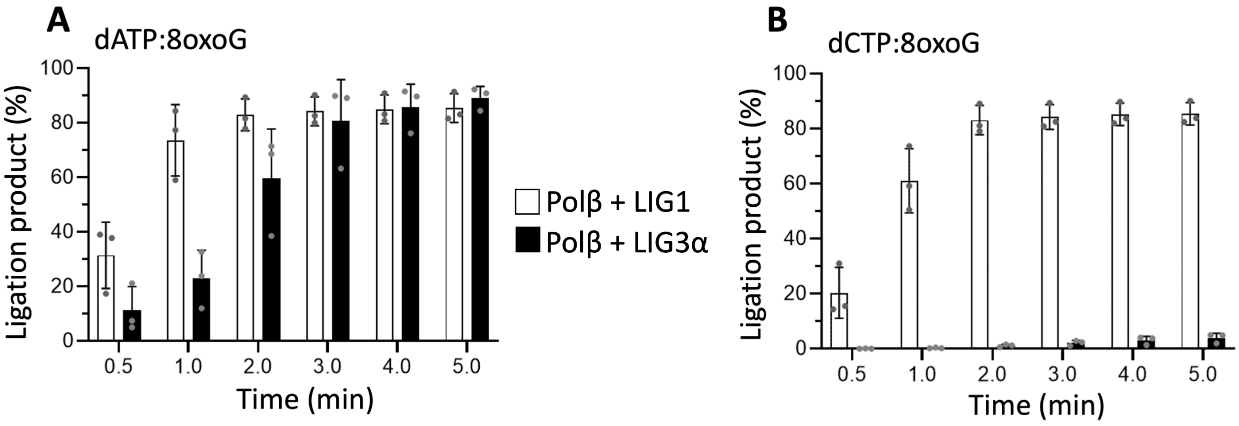

Supplementary Figure 3. Comparison of ligation products by LIG1 *versus* LIG3α after polβ mismatch insertion during 8oxoG bypass. (A-B) Graphs show time-dependent changes in the amount of ligation products after polβ dATP (A) and dCTP (B) mismatch insertions opposite 8-oxoG by LIG1 *versus* LIG3α.

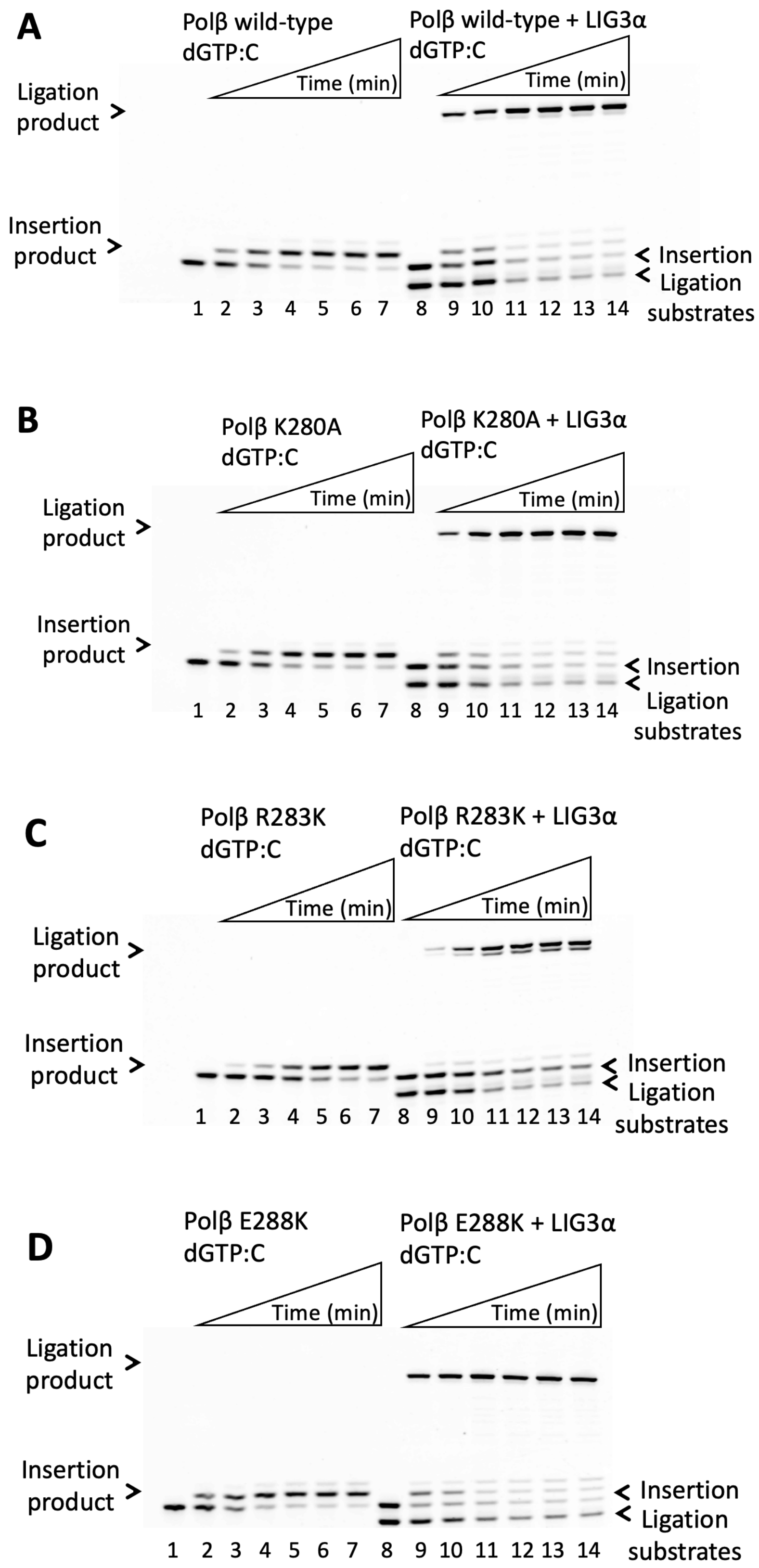
A
Polβ wild-type
dGTP:C
Polβ wild-type + LIG3α
dGTP:C
Time (min)
Time (min)
Ligation product
Insertion product
Insertion
Ligation substrates
1 2 3 4 5 6 7 8 9 10 11 12 13 14
B
Polβ K280A
dGTP:C
Polβ K280A + LIG3α
dGTP:C
Time (min)
Time (min)
Ligation product
Insertion product
Insertion
Ligation substrates
1 2 3 4 5 6 7 8 9 10 11 12 13 14
C
Polβ R283K
dGTP:C
Polβ R283K + LIG3α
dGTP:C
Time (min)
Time (min)
Ligation product
Insertion product
Insertion
Ligation substrates
1 2 3 4 5 6 7 8 9 10 11 12 13 14
D
Polβ E288K
dGTP:C
Polβ E288K + LIG3α
dGTP:C
Time (min)
Time (min)
Ligation product
Insertion product
Insertion
Ligation substrates
1 2 3 4 5 6 7 8 9 10 11 12 13 14

Supplementary Figure 4. Ligation of dGTP:C insertion products by polβ wild-type and mutants. (A-D) Lanes 1 and 8 are the negative enzyme controls of the one nucleotide gap DNA substrate with template C used in polβ insertion and polβ/DNA ligase coupled assays, respectively. Lanes 2-7 are dGTP:C insertion products by polβ wild-type (A), and K280A (B), R283K (C), E288K (D) mutants, and correspond to time points of 0.5, 1, 2, 3, 4, and 5 min. Lanes 9-14 are the ligation of dGTP:C insertion products by polβ wild-type (A), K280A (B), R283K (C), E388K (D) mutants, and correspond to time points of 0.5, 1, 2, 3, 4, and 5 min.

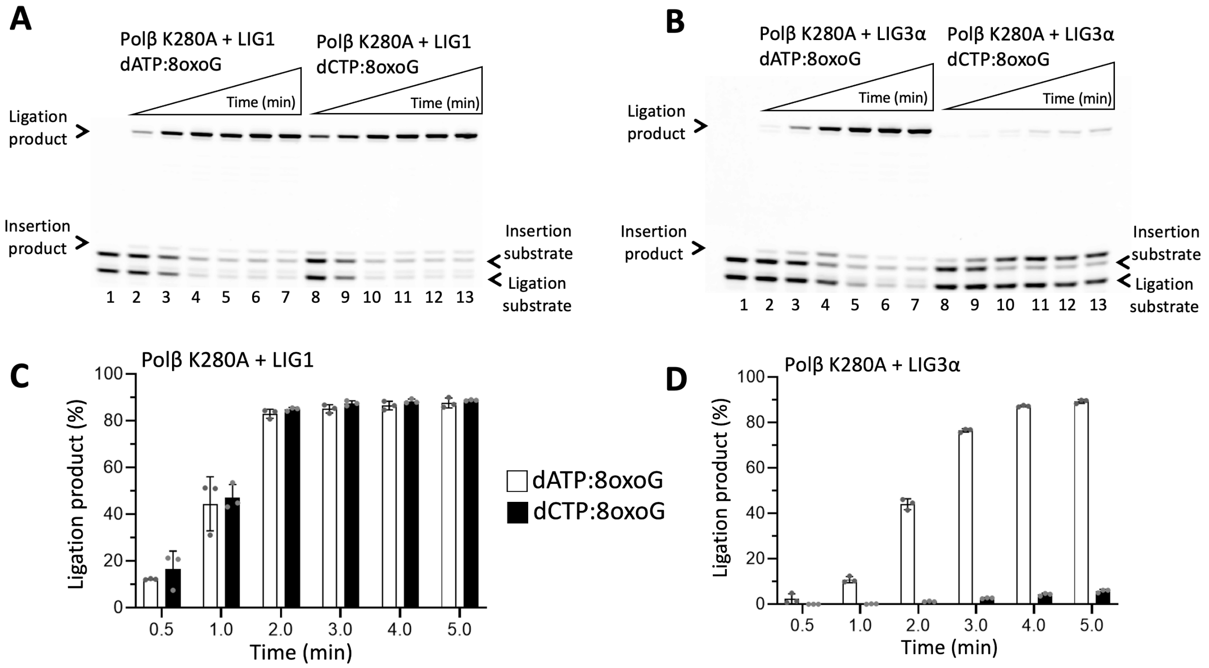

Supplementary Figure 5. Ligation of mismatch insertion products by polβ K280A during 8-oxoG bypass by BER ligases. (A-B) Line 1 is the negative enzyme control of the one nucleotide gap DNA substrate with template 8-oxoG. Lanes 2-7 and 8-13 are the ligation products of polβ K280A dATP and dCTP insertions opposite 8-oxoG, respectively, by LIG1 (A) and LIG3α (B), and correspond to time points of 0.5, 1, 2, 3, 4, and 5 min. **(C-D)** Graphs show time-dependent changes in the amount of ligation products by LIG1 (C) and LIG3α (D). The data represent the average of three independent experiments ± SD.

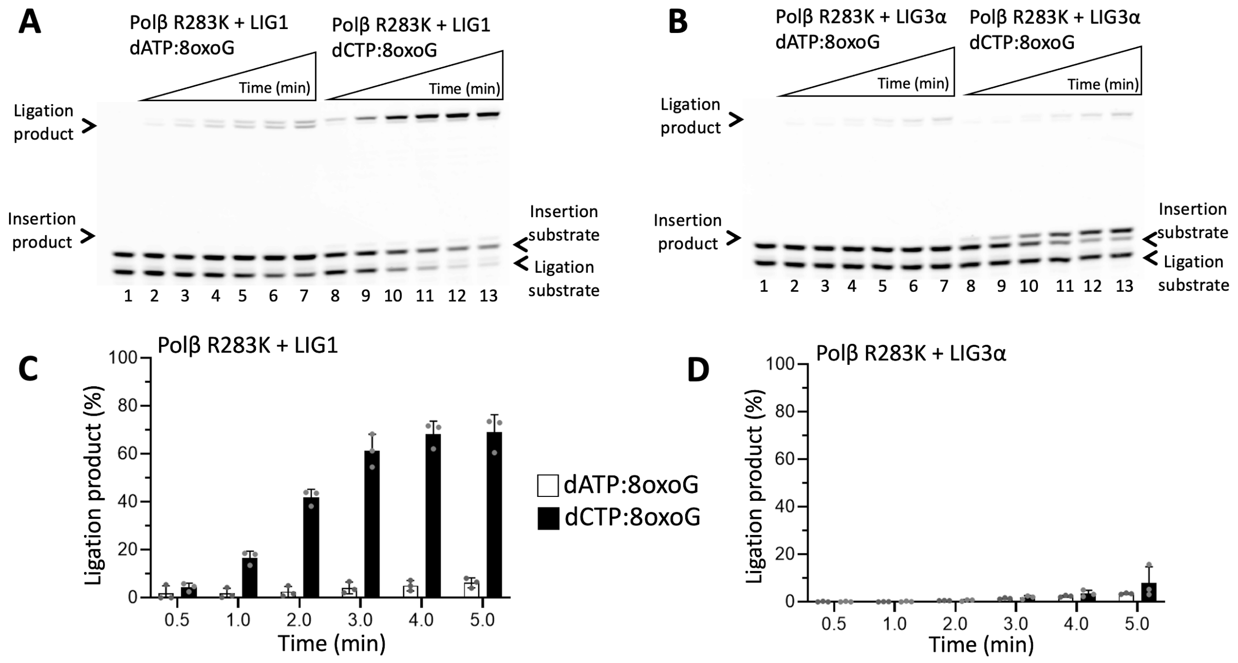

Supplementary Figure 6. Ligation of mismatch insertion products by polβ R283K during 8-oxoG bypass by BER ligases. (A-B) Line 1 is the negative enzyme control of the one nucleotide gap DNA substrate with template 8-oxoG. Lanes 2-7 and 8-13 are the ligation products of polβ R283K dATP and dCTP insertions opposite 8-oxoG, respectively, by LIG1 (A) and LIG3α (B), and correspond to time points of 0.5, 1, 2, 3, 4, and 5 min. **(C-D)** Graphs show time-dependent changes in the amount of ligation products by LIG1 (C) and LIG3α (D). The data represent the average of three independent experiments ± SD.

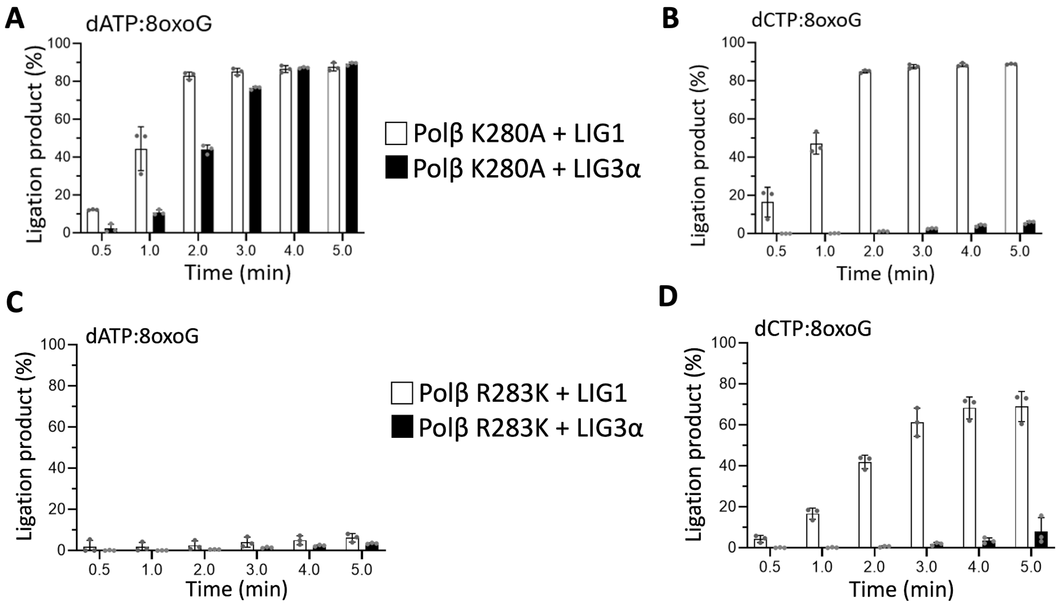

Supplementary Figure 7. Comparison of ligation products by LIG1 *versus* LIG3α after dATP or dCTP insertion opposite template 8-oxoG by polβ active site mutants R280K and R283K. (A-B) Graphs show time-dependent changes in the amount of ligation products after dATP:8-oxoG (A) and dCTP:8-oxoG (B) insertions by polβ K280A mutant to show the comparison between LIG1 and LIG3α. (**C-D)** Graphs show time-dependent changes in the amount of ligation products after dATP:8-oxoG (C) and dCTP:8-oxoG (D) insertions by polβ R283K mutant to show the comparison between LIG1 and LIG3α. The data represent the average of three independent experiments ± SD.

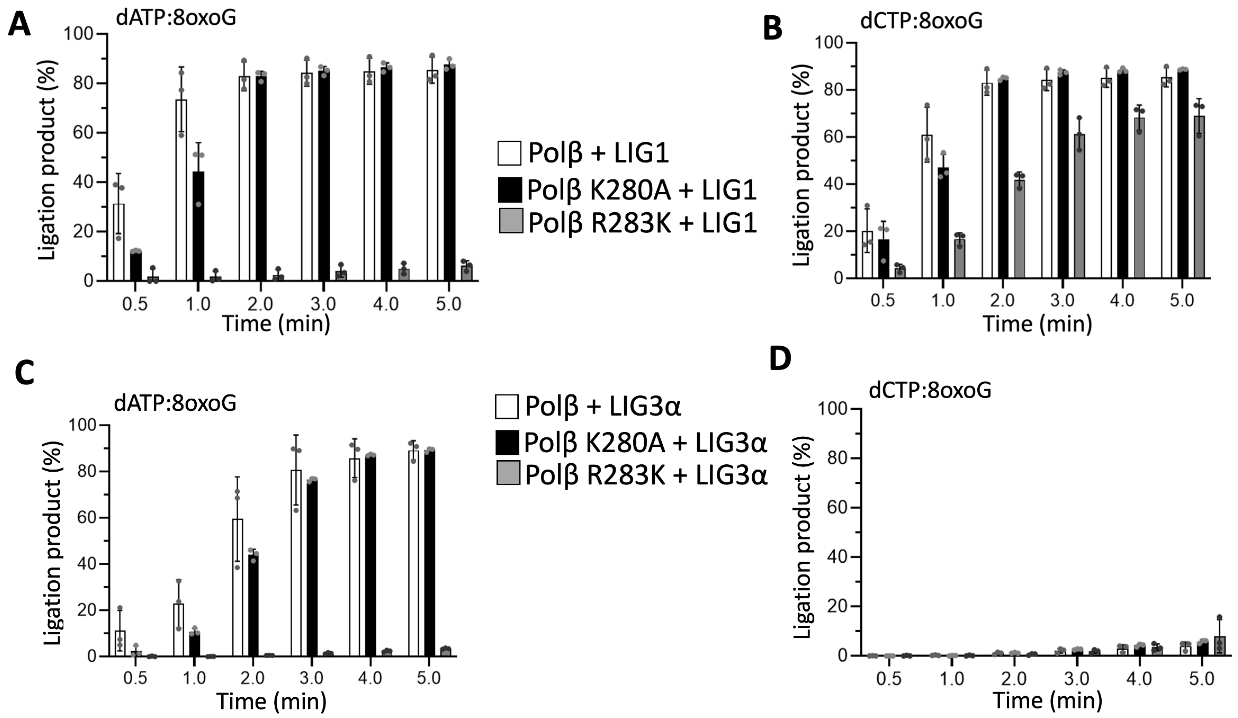

Supplementary Figure 8. Comparison of ligation products by LIG1 or LIG3α after dATP or dCTP insertion opposite template 8-oxoG by polβ wild-type *versus* active site mutants R280K and R283K. (A-B) Graphs show time-dependent changes in the amount of ligation products by LIG1 after dATP:8-oxoG (A) and dCTP:8-oxoG (B) insertions by polβ wild-type and active site mutants K280A and R283K. (**C-D)** Graphs show time-dependent changes in the amount of ligation products by LIG3α after dATP:8-oxoG (C) and dCTP:8-oxoG (D) insertions by polβ wild-type and active site mutants K280A and R283K. The data represent the average of three independent experiments ± SD.

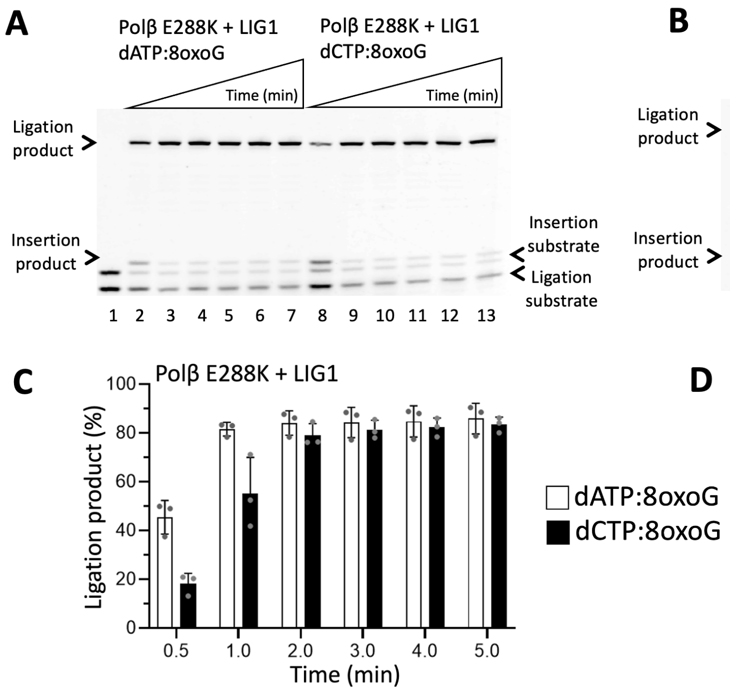

Supplementary Figure 9. Ligation of mismatch insertion products by polβ E288K during 8-oxoG bypass by BER ligases. (A-B) Line 1 is the negative enzyme control of the one nucleotide gap DNA substrate with template 8-oxoG. Lanes 2-7 and 8-13 are the ligation products of polβ E288K dATP and dCTP insertions opposite 8-oxoG, respectively, by LIG1 (A) and LIG3α (B), and correspond to time points of 0.5, 1, 2, 3, 4, and 5 min. **(C-D)** Graphs show time-dependent changes in the amount of ligation products by LIG1 (C) and LIG3α (D). The data represent the average of three independent experiments ± SD.

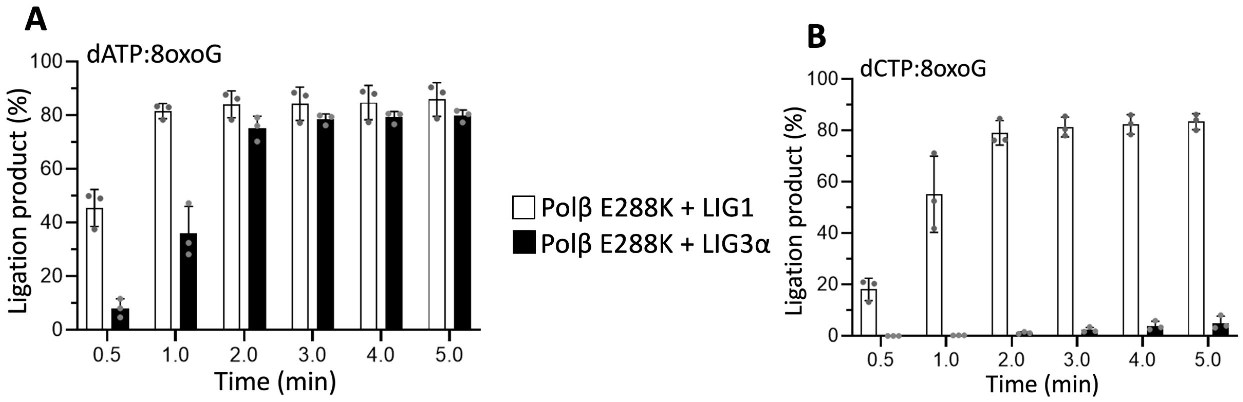

Supplementary Figure 10. Comparison of ligation products by LIG1 *versus* LIG3α after dATP or dCTP insertion opposite template 8-oxoG by polβ cancer-associated variant E288K. (A-B) Graphs show time-dependent changes in the amount of ligation products after dATP:8-oxoG (A) and dCTP:8-oxoG (B) insertions by polβ cancer-associated variant E288K to show the comparison between LIG1 and LIG3α. The data represent the average of three independent experiments ± SD.

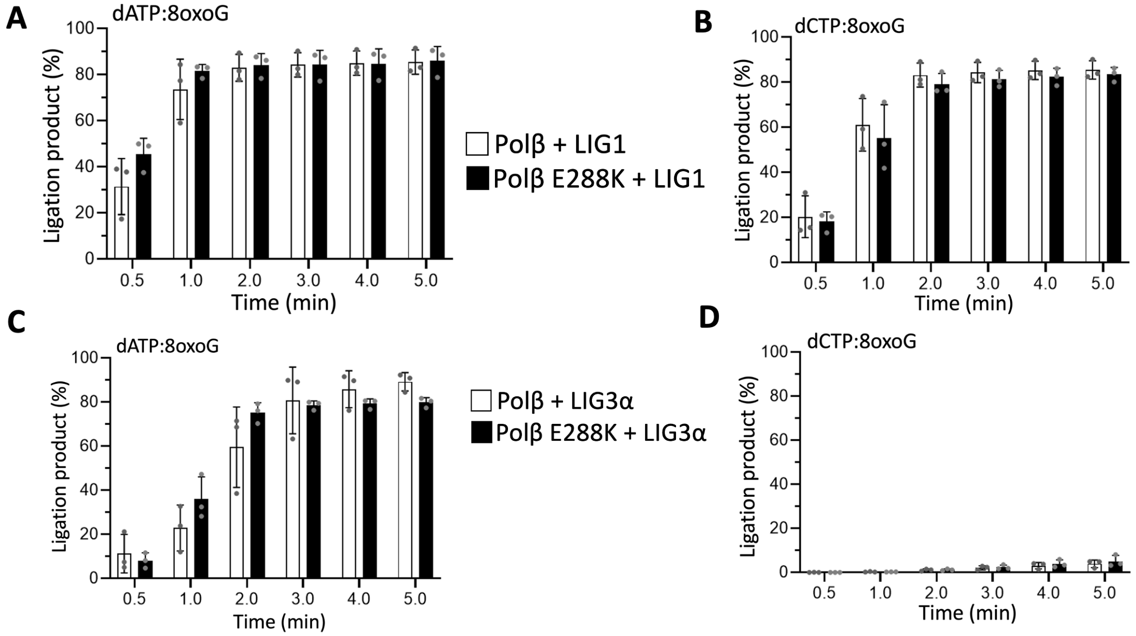

Supplementary Figure 11. Comparison of ligation products by LIG1 or LIG3α after dATP or dCTP insertion opposite template 8-oxoG by polβ wild-type *versus* cancer-associated variant E288K. (A-B) Graphs show time-dependent changes in the amount of ligation products by LIG1 after dATP:8-oxoG (A) and dCTP:8-oxoG (B) insertions by polβ wild-type and cancer-associated variant E288K. **(C-D)** Graphs show time-dependent changes in the amount of ligation products by LIG3α after dATP:8-oxoG (C) and dCTP:8-oxoG (D) insertions by polβ wild-type and cancer-associated variant E288K. The data represent the average of three independent experiments ± SD.

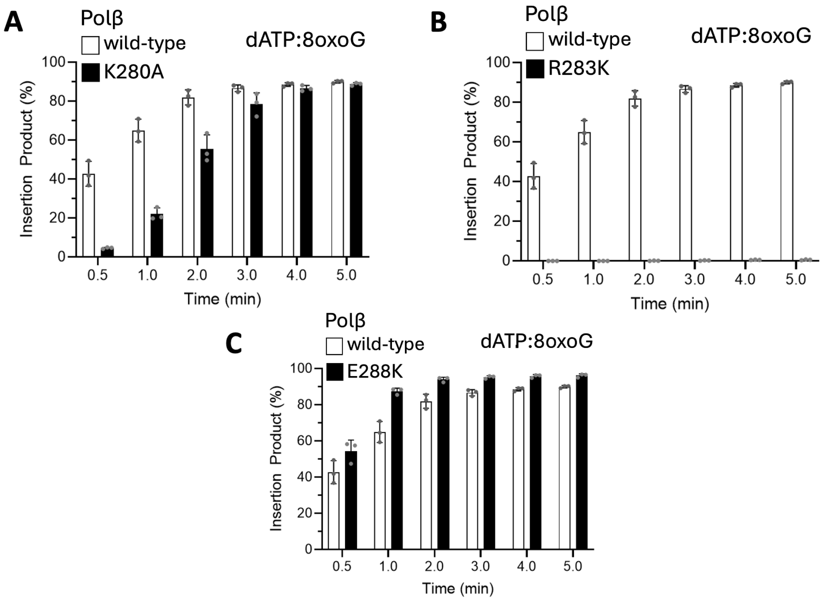

Supplementary Figure 12. Comparisons for dATP mismatch insertion products by polβ wild-type and mutants during 8oxoG bypass. (A-C) Graphs show time-dependent changes in the amount of mismatch insertion products after polβ dATP insertions by polβ wild-type *versus* active site mutants K280A (A), R283K (B), and E288K (C).

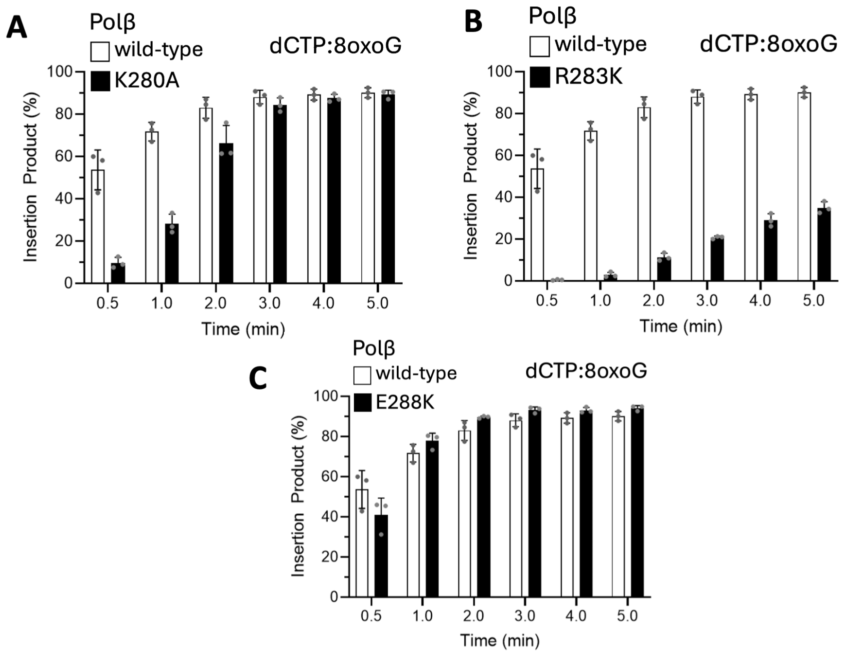

Supplementary Figure 13. Comparisons for dCTP mismatch insertion products by polβ wild-type and mutants during 8oxoG bypass. (A-C) Graphs show time-dependent changes in the amount of mismatch insertion products after polβ dCTP insertions by polβ wild-type *versus* active site mutants K280A (A), R283K (B), and E288K (C).

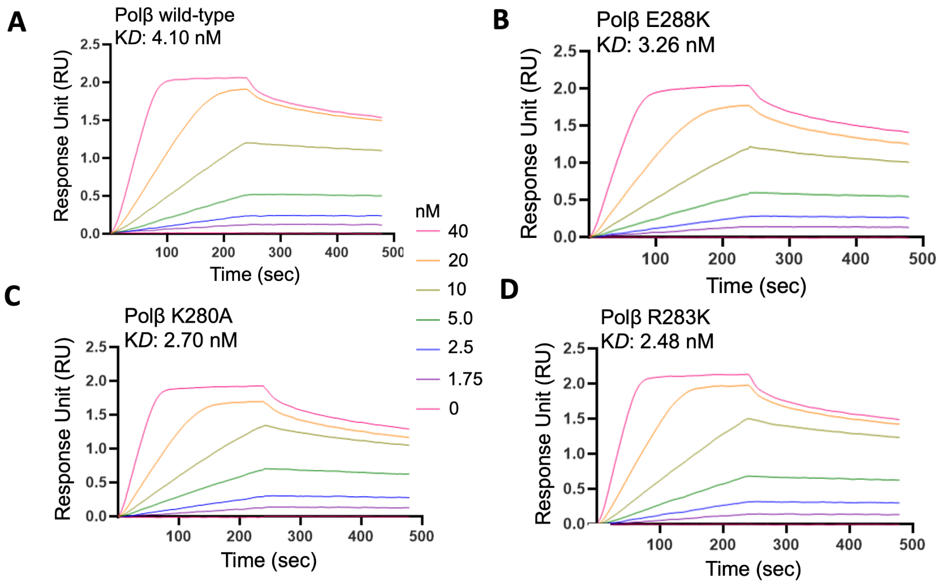

Supplementary Figure 14. Gap DNA binding affinity of polβ wild-type *versus* mutants. (A-D) Real-time binding kinetics of one nucleotide gap DNA are shown for polβ wild-type (A), E288K (B), K280A (C), and R283K (D) mutants. The sensorgrams are shown for the concentrations range of polβ where gap DNA with a biotin label is immobilized on the streptavidin biosensors.

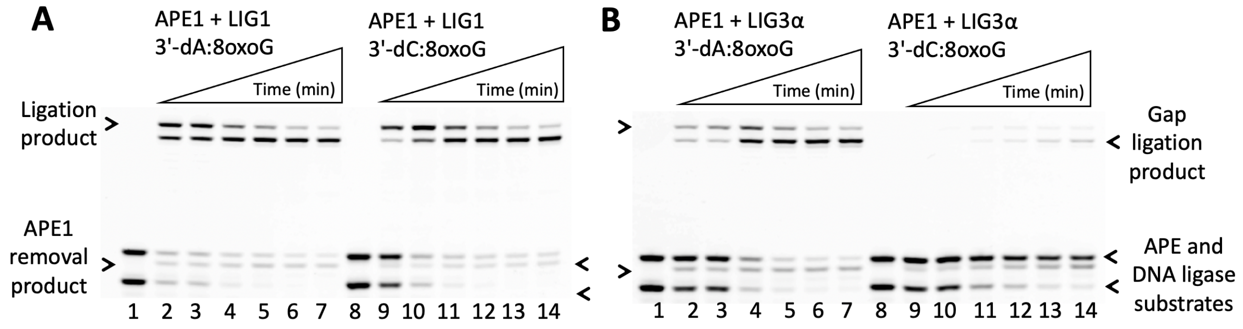

Supplementary Figure 15. Ligation coupled to mismatch removal by APE1 and BER ligases in the presence of nick substrates with 3'-mismatches. (A-B) Lanes 1 and 8 are the negative enzyme controls of the nick DNA substrates with 3'-dA:8-oxoG and 3'-dC:8-oxoG, respectively. Lanes 2-7 and 9-14 are the coupled reaction products showing APE1 mismatch removal and ligation by LIG1 (A) and LIG3α (B) from the nick DNA substrates with 3'-dA:8-oxoG and 3'-dC:8-oxoG, respectively, and correspond to time points of 0.5, 1, 3, 5, 8, and 10 min.

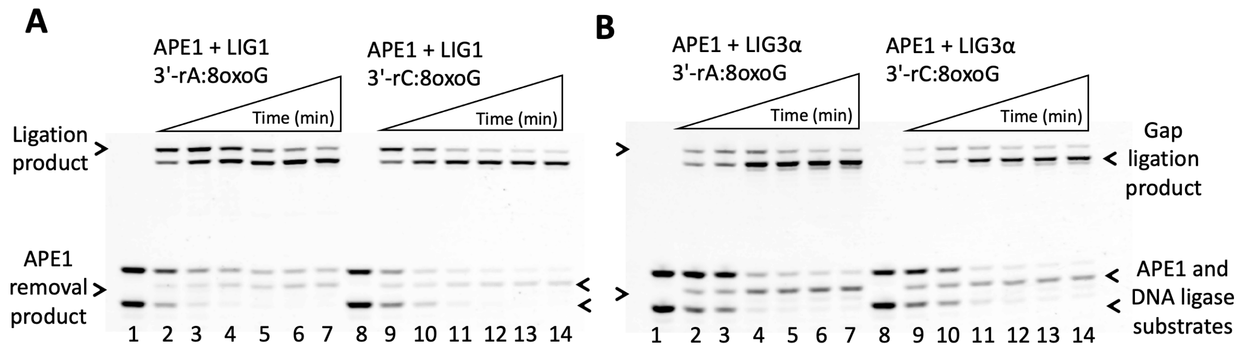

Supplementary Figure 16. Ligation coupled to mismatch removal by APE1 and BER ligases in the presence of nick substrates with 3'-ribonucleotides. (A-B) Lanes 1 and 8 are the negative enzyme controls of the nick DNA substrates with 3'-rA:8-oxoG and 3'-rC:8-oxoG, respectively. Lanes 2-7 and 9-14 are the coupled reaction products showing APE1 ribonucleotide removal and ligation by LIG1 (A) and LIG3α (B) from the nick DNA substrates with 3'-rA:8-oxoG and 3'-rC:8-oxoG, respectively, and correspond to time points of 0.5, 1, 3, 5, 8, and 10 min.

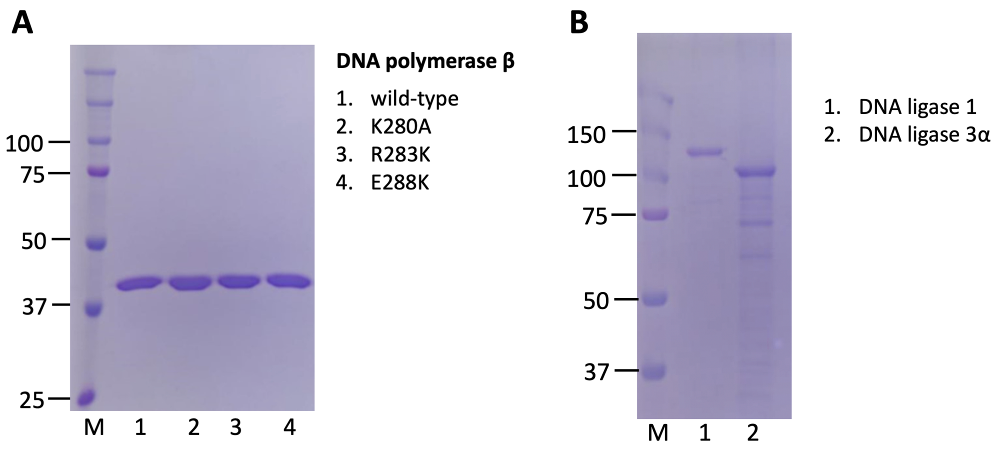

Supplementary Figure 17. The purified proteins used in the study. (A-B) SDS-PAGE gels show the purified proteins for polβ wild-type and mutants K280A, R283K, and E288K (A); LIG1 and LIG3α (B). M represents a Precision Plus Protein Dual Color Standard (10-250 kDa).

DNA Substrates	Sequence
Gap 8oxoG	FAM-5'-CATGGGCGGCATGAACC GAGGCCCATCCTCACC-3'<br>3'-GTACCCGCCGTACTTGGXCTCCGGGTAGGAGTGG-5'
Gap C	FAM-5'-CATGGGCGGCATGAACC GAGGCCCATCCTCACC-3'<br>3'-GTACCCGCCGTACTTGGCCTCCGGGTAGGAGTGG-5'

Supplementary Table 1. Gap DNA substrates used in polβ nucleotide insertion assays. One nucleotide gap DNA substrate with template base C or 8-oxoG and 5'-FAM label were used in the polβ insertion assays to test correct dGTP:C, mismatches dATP:8-oxoG or dCTP:8-oxoG, and rATP:8-oxoG or rCTP:8-oxoG insertions. X presents 8-oxoG and FAM denotes a fluorescent tag. The base at the template position is underlined.

DNA Substrates	Sequence
Gap 8oxoG	FAM-5'-CATGGGCGGCATGAACC GAGGCCCATCCTCACC-3'-FAM<br>3'-GTACCCGCCGTACTTGGXCTCCGGGTAGGAGTGG-5'
Gap C	FAM-5'-CATGGGCGGCATGAACC GAGGCCCATCCTCACC-3'-FAM<br>3'-GTACCCGCCGTACTTGGCCTCCGGGTAGGAGTGG-5'

Supplementary Table 2. Gap DNA substrates used in the coupled assays to measure gap filling by polβ and subsequent nick sealing by BER ligases. One nucleotide gap DNA substrate with template base C or 8-oxoG and FAM labels at 3'- and 5'-ends were used in the coupled assays to test the ligation after polβ correct dGTP:C, mismatches dATP:8-oxoG or dCTP:8-oxoG, and rATP:8-oxoG or rCTP:8-oxoG insertions by LIG1 or LIG3α. X presents 8-oxoG and FAM denotes a fluorescent tag. The base at the template position is underlined.

Nick DNA Substrates	Sequence
3'-dA:8oxoG	FAM-5'-CATGGGCGGCATGAACC**A**GAGGCCCATCCTCACC-3'<br>3'-GTACCCGCCGTACTTGGXCTCCGGGTAGGAGTGG-5'
3'-dC:8oxoG	FAM-5'-CATGGGCGGCATGAACC**C**GAGGCCCATCCTCACC-3'<br>3'-GTACCCGCCGTACTTGGXCTCCGGGTAGGAGTGG-5'
3'-rA:8oxoG	FAM-5'-CATGGGCGGCATGAACC**rA**GAGGCCCATCCTCACC-3'<br>3'-GTACCCGCCGTACTTGGXCTCCGGGTAGGAGTGG-5'
3'-rC:8oxoG	FAM-5'-CATGGGCGGCATGAACC**rC**GAGGCCCATCCTCACC-3'<br>3'-GTACCCGCCGTACTTGGXCTCCGGGTAGGAGTGG-5'

Supplementary Table 3. Nick DNA substrates used in APE1 exonuclease assays. X presents 8-oxoG and FAM denotes a fluorescent tag. A ribonucleotide (rA or rC) or a mismatched base (dA or dC) at the 3'-end of nick DNA substrates are shown as bold and the template 8-oxoG is underlined.

Nick DNA Substrates	Sequence
3'-dA:8oxoG	FAM-5'-CATGGGCGGCATGAACC**A**GAGGCCCATCCTCACC-3'-FAM<br>3'-GTACCCGCCGTACTTGGXCTCCGGGTAGGAGTGG-5'
3'-dC:8oxoG	FAM-5'-CATGGGCGGCATGAACC**C**GAGGCCCATCCTCACC-3'-FAM<br>3'-GTACCCGCCGTACTTGGXCTCCGGGTAGGAGTGG-5'
3'-rA:8oxoG	FAM-5'-CATGGGCGGCATGAACC^{r}**A**GAGGCCCATCCTCACC-3'-FAM<br>3'-GTACCCGCCGTACTTGGXCTCCGGGTAGGAGTGG-5'
3'-rC:8oxoG	FAM-5'-CATGGGCGGCATGAACC^{r}**C**GAGGCCCATCCTCACC-3'-FAM<br>3'-GTACCCGCCGTACTTGGXCTCCGGGTAGGAGTGG-5'

Supplementary Table 4. Nick DNA substrates used in the coupled assays to test APE1 exonuclease removal coupled to ligation by BER ligases. X presents 8-oxoG and FAM denotes a fluorescent tag. A ribonucleotide (rA or rC) or a mismatched base (dA or dC) at the 3'-end of nick DNA substrates are shown as bold and the template 8-oxoG is underlined.

DNA substrate	Sequence
Gap C	5'-CATGGGCGGCATGAACC ^{P}GAGGCCCATCCTCACC-3'-Bio<br>3'-GTACCCGCCGTACTTGGCCTCCGGGTAGGAGTGG-5'

Supplementary Table 5. One nucleotide gap DNA substrate used in BLI assays to analyze DNA binding affinity of polβ wild-type and mutants. The position of template base is undelined. Bio stands for biotin-labeled at 3'-end of DNA substrate.
